# Artificial intelligence for TNM staging in NSCLC: a critical appraisal of segmentation utility in [^1^⁸F]FDG PET/CT

**DOI:** 10.1007/s00259-025-07677-2

**Published:** 2025-11-23

**Authors:** Maurice M. Heimer, Jakob Dexl, Johanna Ta, Ricarda Ebner, Felix L. Herr, Leon Orasanin, Katharina Jeblick, Lisa C. Adams, Lalith K. Shiyam Sundar, Amanda Tufman, Rudolf A. Werner, Gabriel Sheikh, Jens Ricke, Michael Ingrisch, Matthias P. Fabritius, Clemens C. Cyran

**Affiliations:** 1 Department of Radiology, LMU University Hospital, LMU Munich, Munich, Germany; 2 Munich Center for Machine Learning (MCML), Munich, Germany; 3 Comprehensive Pneumology Center (CPC-M), Member of the German Center for Lung Research (DZL), Munich, Germany; 4 Department of Radiology, TUM University Hospital, TU Munich, Munich, Germany; 5 Department of Medicine V, LMU University Hospital, LMU Munich, Munich, Germany, and Bavarian Center for Cancer Research (BZKF), Munich, Germany; 6 Department of Nuclear Medicine, LMU University Hospital, LMU Munich, Munich, Germany; 7 The Russell H. Morgan Department of Radiology and Radiological Sciences, Division of Nuclear Medicine and Molecular Imaging, Johns Hopkins University School of Medicine, Baltimore, MD USA

**Keywords:** Artificial intelligence, [¹⁸F] FDG PET/CT, Non-small cell lung cancer, External validation, Task-specific evaluation

## Abstract

**Purpose:**

This study aims to investigate whether a diagnostic AI model can effectively support lesion detection and staging in non-small cell lung cancer (NSCLC) [^1^⁸F]FDG PET/CT studies, focusing on the distinction between technical segmentation accuracy and clinically meaningful performance.

**Methods:**

In this retrospective single-centre study, [^1^⁸F]FDG PET/CT scans from 306 treatment-naïve NSCLC patients were reviewed with reference to multidisciplinary team decisions. Tumour lesions were manually segmented for reference and compared with predictions from the top-performing algorithm of the autoPET III challenge. Quantitative segmentation metrics were calculated, and lesion-level errors were assessed for impact on patient-level TNM and UICC staging.

**Results:**

The algorithm achieved a mean Dice Similarity Coefficient (DSC) of 0.64. Lesion-level sensitivity was 95.8% across all patients, with a precision of 87.5%. False positive M-category lesions (*n* = 196) occurred as most frequent error. Of all false positives, 35.7% were benign and 34.7% non-oncologic pathologies. UICC staging matched ground truth in 207/306 patients, with most discordances due to upstaging (88/306).

**Conclusion:**

Clinically driven metrics and cause-based error analysis offer valuable insight into AI segmentation performance. The evaluated model showed excellent lesion sensitivity but a tendency towards systematic overprediction across TNM categories. On a lesion level M-stage false positives and undersegmentation in the hilar region emerged as the main driver of clinically relevant upstaging. Despite promising lesion detection sensitivity, only 67.7% UICC-stagings were accurate using AI masks, indicating that diagnostic AI may support, though not yet replace, manual lesion evaluation in NSCLC [^1^⁸F]FDG PET/CT.

**Supplementary Information:**

The online version contains supplementary material available at 10.1007/s00259-025-07677-2.

## Introduction

The integration of artificial intelligence (AI) into oncological imaging, particularly in hybrid imaging such as [^18^F]fluorodeoxyglucose positron emission tomography/computed tomography (FDG PET/CT), has introduced new possibilities for automatic assessment of tumour burden [1–4]. Community-driven initiatives such as autoPET and HECKTOR have demonstrated the potential of U-Net–based models for FDG-avid lesion delineation and have established benchmarks across cancer types [5–9].

While accurate image segmentations are essential for deriving quantitative imaging biomarkers, such as total metabolic tumour volume (TMTV) or total lesion glycolysis (TLG) [10–12], manual segmentations remain challenging [13]. Segmentation models are commonly evaluated using voxel-level overlap metrics such as the Dice Similarity Coefficient (DSC). However, such metrics may not fully capture the clinical relevance of systematic bias or prediction errors with potential impact at the lesion- or patient-level [2, 14–16].

Non-small cell lung cancer (NSCLC) staging provides a particularly relevant use case. Accurate tumour, node, metastasis (TNM) staging requires more than volume estimation but also the accurate detection of individual oncological lesions, precise boundary delineation and differentiation of non-oncological pathology [17, 18]. The clinical impact of segmentation errors is not uniform: some errors may be inconsequential, particularly in advanced metastatic disease, whereas others may lead to stage migration and potential mismanagement. For example, missing a solitary distant metastasis could result in clinically relevant downstaging and might lead to curative-intent surgery instead of appropriate palliative treatment or an oligometastatic management concept.

In this study, we evaluate the clinical utility of the highest-performing algorithm from the autoPET III challenge [19] for automated TNM staging in treatment-naïve NSCLC patients. Using 306 [^1^⁸F]FDG PET/CT scans, we compare AI-based and reference staging, assess the clinical significance of segmentation errors and develop an error taxonomy to characterise common failure modes. This work is situated within the broader effort to move beyond conventional overlap metrics and towards task-aware evaluations that reflect clinical relevance. In line with emerging guidelines such as RELAINCE [15], it addresses the need for clinically meaningful performance measures and highlights the importance of transparent, interpretable integration of AI into medical imaging [20].

The aim of this work is to determine whether state-of-the-art segmentation can support clinically meaningful staging decisions beyond conventional overlap metrics.

## Material and methods

This single centre retrospective study has been approved by the institutional review board (*22–0416*), the need for written informed consent was waived.

### Data

A total of 1,947 patients with lung cancer were treated at the Munich Lung Cancer Centre between January 2018 and December 2021. Of these, 402 patients with histologically confirmed NSCLC underwent pre-therapeutic whole-body [^18^F]FDG PET/CT imaging with contrast-enhanced CT. To enable consistent manual validation and quality control, a randomly selected subset of 306 patients was analysed. Relevant clinical information, tumour histology and imaging acquisition information were extracted from electronic health records and summarized in Table 1.

**Table 1:**
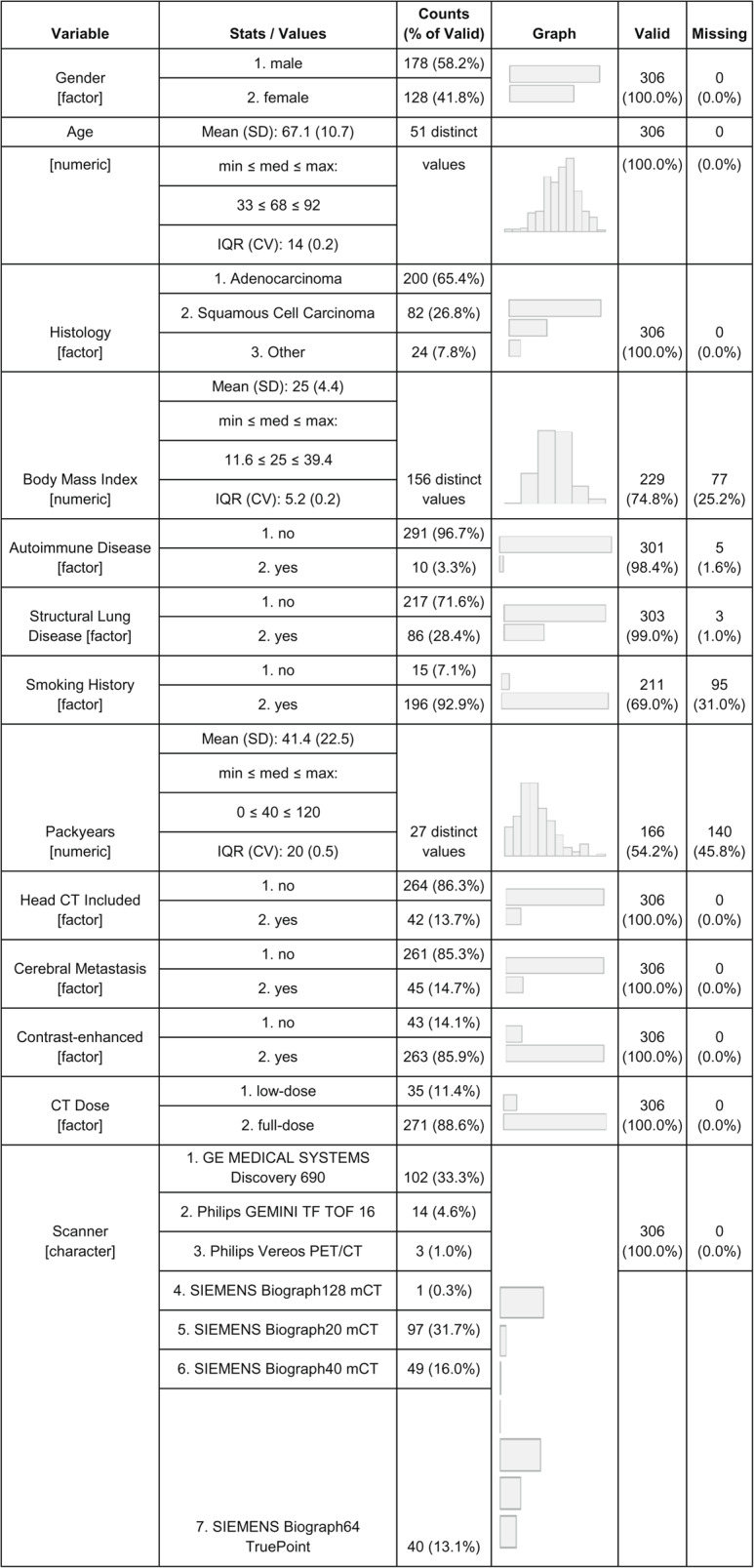
Baseline characteristics of the study population and imaging protocols*.* Values are presented as absolute numbers with percentages referring to the proportion of valid (non-missing) cases for each variable. Continuous variables are reported as mean ± standard deviation (SD) along with range, median, and interquartile range (IQR) where applicable

Diagnostic scans were acquired in accordance with the European Association of Nuclear Medicine (EANM) guidelines for tumour imaging, ensuring standardized acquisition protocols and consistent image quality. Attenuation-corrected PET images were normalized to standardized uptake values (SUV) to enable semi-quantitative analysis. Overall, *n* = 263 (85.9%) of studies were performed with CT contrast agent in venous phase. A low-dose PET/CT protocol was applied when a full-dose CT had already been acquired as part of the prior diagnostic work-up (11.4%, *n* = 35). Extension of the whole-body PET/CT to include the head was performed in cases where cranial magnetic resonance imaging (MRI) was contraindicated or otherwise not feasible (13.7%, *n* = 42). Scanner specific imaging protocols are found in supplementary Table 1.

All studies were reviewed by two hybrid imaging experts (M.M.H. and M.P.F., with 5 and 9 years of experience in hybrid imaging, respectively) in consensus, using a CE-certified structured and software-based reporting platform (*Mint Lesion™*, Mint Medical, Heidelberg, Germany) [21]. For ground truth, only tumour burden was defined as true lesions, excluding physiological uptake and non-oncologic findings. Lesions were annotated and staged according to the 9th edition of the TNM classification system [17], with additional input from multidisciplinary tumour board decisions to aid interpretation of equivocal or ambiguous findings. All lesions were individually segmented by hand in standard attenuation-corrected PET reconstructions and soft-kernel CT images to reflect anatomic tumour burden. TMTV was derived as the sum of all segmented lesions in individual patients.

### Automated tumour quantification

Additionally, segmentations were generated using the autoPET III challenge highest-performing algorithm [19] which aimed at multitracer and multicentre generalization. The model builds on the nnUNet framework with a residual encoder layer architecture and incorporates key enhancements such as large-scale pretraining, misalignment augmentation, and organ-aware segmentation. It processes both PET and CT inputs to improve lesion detection and provide anatomical context. Training data included 1014 [^1^⁸F]FDG and 597 [^64^Ga]/[^1^⁸F]Prostate specific membrane antigen (PSMA) PET/CT scans from LMU Hospital Munich and the University Hospital Tuebingen across four cancer types (lymphoma, lung cancer, melanoma and prostate cancer). A more detailed description of the AI model is provided by Rokuss et al. [19]. There was no overlap between the training and testing data of the challenge and the data used in this study.

Voxel-wise segmentation was evaluated against ground truth segmentations using autoPET challenge metrics including mean DSC, false negative volume (FNV), and false positive volume (FPV) [7]. Ground truth and predicted tumour volumes were compared using Bland–Altman analysis.

### Qualitative error analysis

After a washout period of more than one year, the same two hybrid imaging experts independently re-evaluated the AI-generated segmentation masks, which were overlaid on the original [^1^⁸F]FDG PET/CT studies using a custom-built 3D Slicer extension [22]. This extension displayed resampled CT and corresponding SUV images side by side, with the predicted segmentation masks superimposed. The tool included functionality for structured reporting, maximum intensity projection, image windowing, and lesion size measurement.

As a prerequisite for subsequent clinical evaluation, each delineated, predicted segmentation was considered a distinct lesion, irrespective of size. Segmentation errors were stratified by T-, N-, and M-category and further annotated according to the most plausible clinical context or presumed underlying uptake pattern, including physiological uptake, unequivocally benign aetiologies, and pathologic non-oncologic causes. True positives were strictly defined as lesions interpreted as malignant by both readers, while false positives and false negatives encompassed all other discrepancies between predictions and ground truth interpretations. Perceived detection sensitivity was defined as the number of true positive lesions divided by the total number of ground truth positive lesions. The positive predictive value (precision) was defined as the number of true positives divided by the sum of true and false positives.

### Classification performance analysis

On a patient level, clinical staging was repeated using AI-predicted lesions with CT overlays, following the 9th edition of the TNM classification system [17]. Segmentation errors were carried forward into the staging assessment. A higher AI-predicted TNM stage compared to ground truth was defined as upstaging, and a lower stage as downstaging. T-stage lesion size and staging-relevant infiltration patterns were derived from the predicted segmentations, with anatomical correlation provided by CT overlays. The occurrence and impact of over- and undersegmentation on TNM staging were systematically recorded.

### Evaluation of UICC stage migration

Clinical TNM classifications were mapped to UICC stages according to the 9th edition of the AJCC staging manual. Stage concordance between ground truth and AI-derived segmentations was evaluated, with additional assessment of migration across predefined clinical decision boundaries. A higher AI-predicted UICC stage compared to ground truth was defined as upstaging, and a lower stage as downstaging. Simplified clinical decision boundaries were defined between UICC stages IB/IIA and IIIC/IVA to capture the clinical implications of stage migration and their potential influence on treatment allocation. The IB/IIA threshold reflects the transition from primarily surgical management with selective adjuvant therapy to a stage where adjuvant systemic treatment is routinely indicated, while the IIIC/IVA threshold marks the shift from potentially curative multimodal approaches to predominantly palliative systemic strategies.

## Results

### Tumour quantification

**Fig. 1:**
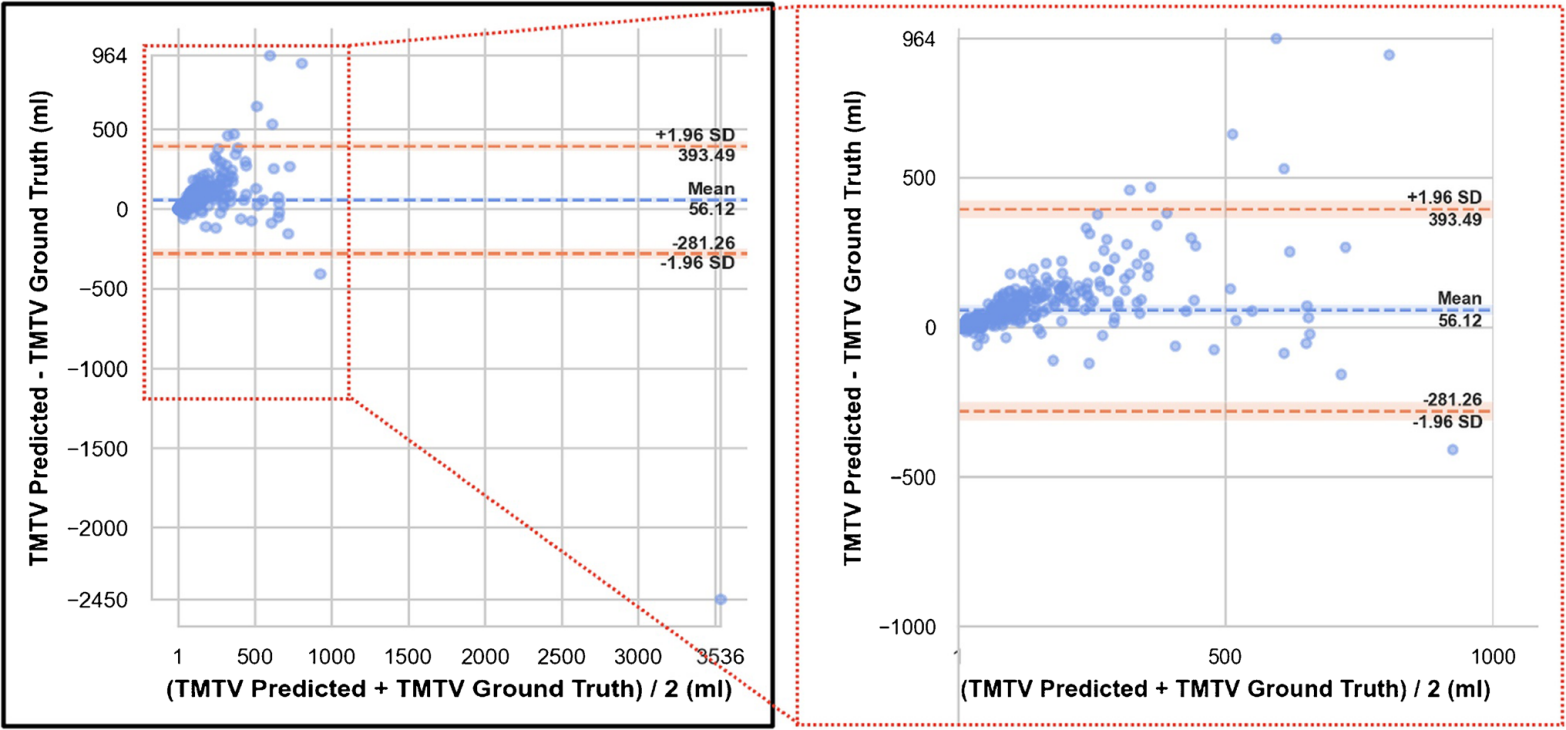
Bland-Altman analysis comparing predicted and ground truth total metabolic tumour volume (TMTV). The solid blue line indicates the mean difference (bias), compared to the limits of agreement (dashed orange lines; mean ± 1.96 standard deviations). Most data points lie within these limits, indicating acceptable agreement between the model predictions and expert-derived TMTV values. Note the single most relevant outlier (bottom right) with significant false positive volume was retrospectively explained by false positive prediction of the total liver due to a weight-based standardization error

The mean difference between predicted and ground truth volumes was 56.1 mL, indicating a volumetric overestimation by the AI model. Limits of agreement ranged from -281.3 mL to + 393.4 mL. Mean FNV was 0.94 ml, with a FPV of 9.49 ml. The mean DSC was 0.64 and closely aligns with the benchmark value of 0.66 reported in the AutoPET III challenge [19]. An exploratory visualization of model performance across patient and imaging subgroups is provided in supplementary Fig. 1. Most discrepancies occurred in patients with high tumour burden, where a few large outliers accounted for substantial differences. These results indicate good overall agreement, with minimal systematic bias and variance primarily driven by individual extreme cases as shown in Fig. 1.

### Qualitative error analysis

**Fig. 2:**
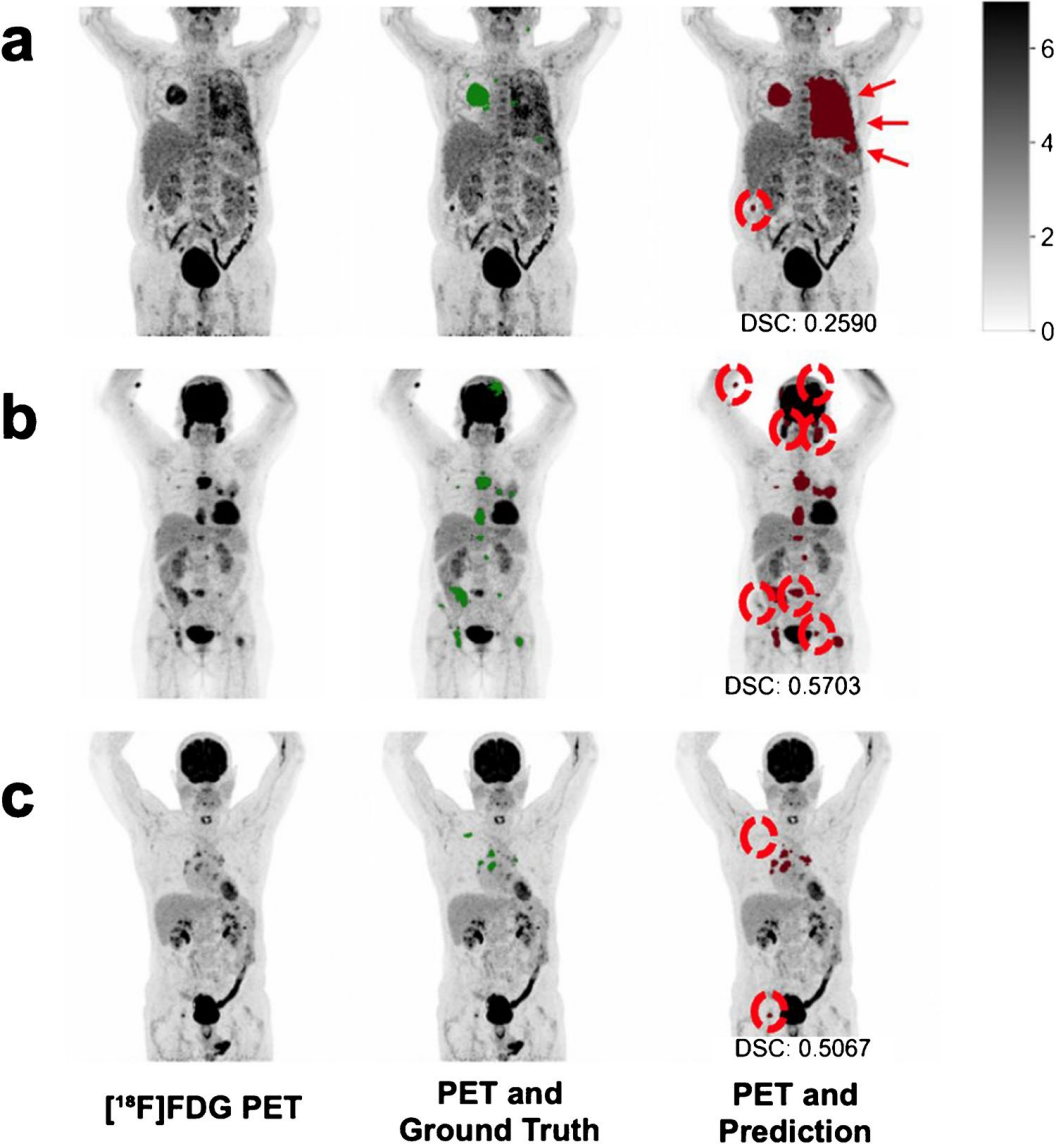
Graphical comparison of predicted segmentations to ground truth in representative cases with relatively low DSC. Maximum intensity projection PET reconstructions are used as base image (left). Predictions (right) are compared to ground truth segmentations (middle). Relevant differences are highlighted by red dotted circles. Panel **a** showcases a patient with linear pleural carcinosis in which the predicted lesions appeared more accurate considering the linear distension compared to handcrafted CT-guided segmentations. However, in the same patient a small false positive segmentation in the colon led to upstaging. **b** demonstrates a case with acceptable resolution of multiple bone metastasis, however false positives of the parotid glands, paravasation and a bladder diverticulum led to upstaging. **c** shows a case with a false negative small lepidic adenocarcinoma primary tumour (T1mi) that demonstrated no significant FDG uptake. Also, a false positive of the right hip was primarily explained by degenerative changes

**Fig. 3:**
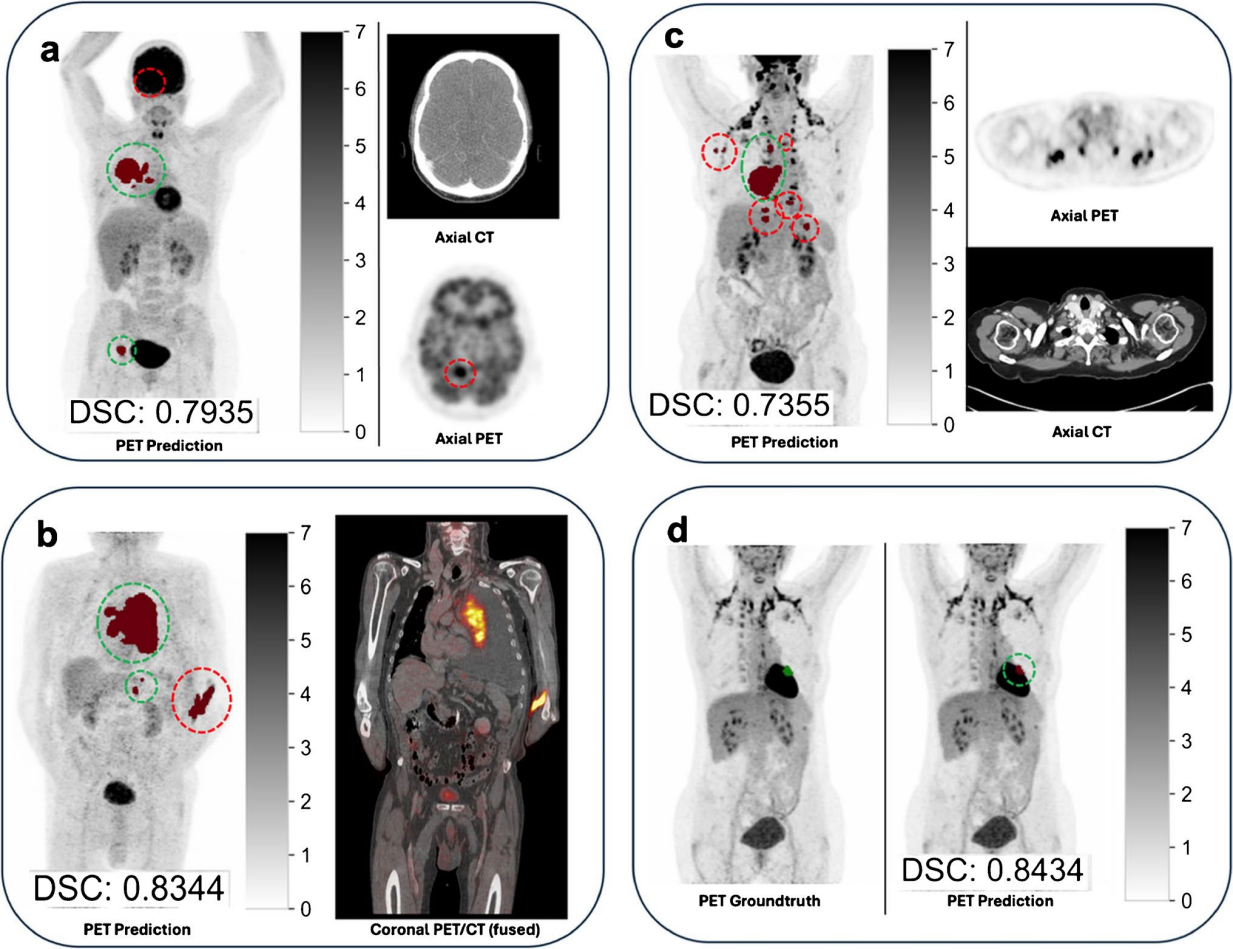
Representative prediction pitfalls in cases with high DSC. Relevant false positives are highlighted by red dotted circles, true positives in green. **a** demonstrates a case with a false negative right occipital lobe metastasis, resulting in downstaging—the bottom right attenuated-corrected PET was windowed to SUV centre = 4.5, width = 9 to improve lesion contrast to noise. **b** showcases a patient with extensive left cubital paravasation, which is easily discerned as a benign cause of uptake. The extensive locoregional and hepatic tumour burden was adequately predicted. **c** and **d** show two cases with extensive brown adipose tissue in typical locations. While some lesions are falsely predicted in **c**, there are no false positives in **d** which highlights that the algorithm’s evaluation of the same uptake pattern can result in different outcomes

At lesion level, the algorithm demonstrated high overall perceived detection sensitivity and precision of 95.8% and 87.5%, respectively. T-stage detection was excellent, with a detection sensitivity of 96.7% (411/425 lesions) and a precision of 94.9% (411/433 lesions). T-stage false positives were primarily attributable to inflammatory changes (*n* = 18) and areas of dystelectasis (*n* = 4). N-stage detection similarly achieved high accuracy, with a detection sensitivity of 95.9% (828/853 lesions) and a precision of 95.7% (828/865 lesions). For M-stage lesions, detection sensitivity was also excellent at 94.8% (548/578 lesions); however, precision was lower at 73.7% (548/744 lesions), reflecting a higher rate of false-positive detections. Detailed qualitative analysis of M-category errors revealed that, while 29.6% (*n* = 58) of false positives were due to physiologic uptake, the majority (70.4%, *n* = 138) were associated with benign or non-malignant pathologies considered false positives only within the strict context of our staging task. Contralateral pulmonary inflammation or dystelectasis were the second most common pathological cause of M-stage false positives, accounting for 7.1% (15/196) of all such cases. A detailed assessment of error types is provided in Table 2. Representative examples of misclassifications are shown in Figs. 2 and 3.

**Table 2:** Detailed error analysis of M-stage lesions with reference to expert reading and multidisciplinary team consensus

False Positives (*n* = 196)		
Physiologic Uptake (*n* = 58)	Benign Cause/Lesion (*n* = 70)	Pathologic Cause/Lesion (*n* = 68)
Gastrointestinal peristalsis (*n* = 15)	Musculoskeletal degenerative disease (*n* = 39)	Rib fracture (*n* = 15)
Physiologic urinary excretion (*n* = 13)	Distant unspecific lymph node (*n* = 10)	Pulmonary inflammation (*n* = 14)
Brown adipose tissue (*n* = 10)	Unspecific prostate uptake (*n* = 8)	Vertebral body fracture (*n* = 7)
Bone activation (*n* = 8)	FDG paravasation or contamination (*n* = 7)	Thyroid gland uptake (*n* = 7)
Oesophageal peristalsis (*n* = 5)	Benign breast lesions (*n* = 3)	Kyphoplasty-associated uptake (*n* = 5)
Muscle activation (*n* = 4)	Drainage associated uptake (*n* = 2)	Liver lesion (*n* = 5)
Anal sphincter (*n* = 2)	Unspecific uterine uptake (*n* = 1)	Parotid inflammation/mass (*n* = 5)
Renal medulla (*n* = 1)		Adrenal adenoma (*n* = 4)
		Skin lesion (*n* = 3)
		Colon polyp (*n* = 3)
False Negatives (*n* = 30)		
Contralateral pulmonary (*n* = 11)		
Liver (*n* = 6)		
Brain (*n* = 4)		
Adrenal gland (*n* = 3)		
Distant lymph node (*n* = 2)		
Peritoneal (*n* = 1)		
Bone (*n* = 1)		
Pleura (*n* = 1)		
Kidney (*n* = 1)

### Classification performance analysis

**Fig. 4:**
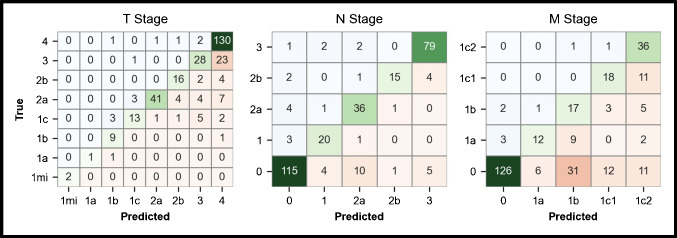
TNM classification and migration between prediction and ground truth. Shown are confusion matrices for T stage (left), N stage (middle), and M stage (right). Rows represent the ground truth as determined by hybrid imaging expert/multidisciplinary team staging, while columns indicate the predicted stage using predicted segmentations. Correct classifications appear on the diagonal, with darker green shades indicating higher accuracy. Off-diagonal elements represent misclassifications, color-coded by frequency (blue = downstaging, red = upstaging)

Clinical TNM staging based on predicted segmentations achieved strong concordance with ground truth staging, as demonstrated in Fig. 4. The analysis demonstrated highest accuracy in predicting N-stage (265/306 patients), as compared to T-stage (240/306 patients) and M-stage (209/306 patients). Prediction-based category downstaging was infrequent in T-stage (12/306 patients), N-stage (16/306 patients) and M-stage (8/306 patients). In contrast, upstaging was observed more frequently in 55/306, 26/306 and 90/306 patients for the corresponding stages. Overall, undersegmentation was observed in 119/306 patients in the hilar region which resulted in 34 cases T- and N- stage classification errors. In contrast, no case of oversegmentation was observed. Inaccurate size segmentation of primary tumours resulted in 13/306 cases of T-stage migration. In 21/306 patients false positive, and in 6/306 patients false negative lesions had no impact on correct TNM classification.

### Evaluation of UICC stage migration

**Fig. 5:**
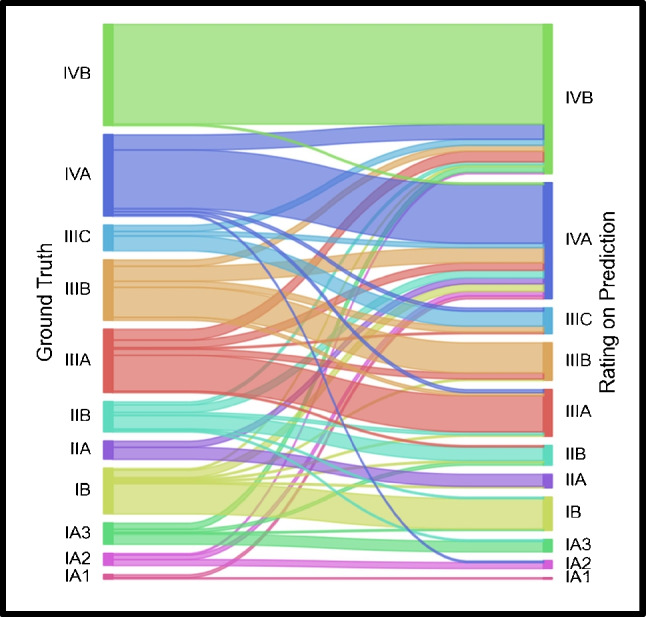
TNM-based UICC classification stage migration derived from ground truth vs. model-based segmentations*.* The Sankey diagram visualizes stage group transitions between ground truth staging (left nodes, based on manual expert segmentations) and predicted staging (right nodes, based on automated model output). Each coloured fluvial represents the number of patients mapped from one stage group to another, highlighting concordant predictions (straight paths) as well as cases of upstaging and downstaging (divergent paths)

UICC staging was concordant in 207 patients (67.4%) between ground truth and AI-derived segmentations, as shown in Fig. 5. A significant trend towards upstaging was observed (88/306 patients) compared with downstaging (11/306 patients). A total of 26 observed stage migrations occurred between adjacent stages, resulting in higher agreement of 76.1% (234/306 patients) when assessed within predefined simplified clinical stage boundaries, with lower rates of significant upstaging (66/306 patients) and downstaging (7/306 patients), as shown in Table 3. Notable shifts included upward reclassification from stages IIIA and IIIB to IVA, frequently driven by overestimation and false positive lesion predictions in areas of ambiguous nodal or distant uptake. Downstaging was less common and primarily involved patients with intermediate-stage disease.

**Table 3:** Impact levels of stage migration on treatment decisions based on simplified clinical decision boundaries (UICC IB/IIA and IIIC/IVA). High impact reflects migration across both decision boundaries, moderate impact denotes migration across one boundary within the same treatment modality, low impact indicates minimal therapeutic consequences, and no impact represents concordant staging within the same boundary

		Count (%)		
Impact Level	Definition	Up Staging	Down Staging	Potential impact on treatment
High	Discrepancy across two boundaries	*n* = 16 (5.2%)	*n* = 1 (0.3%)	Significant change in treatment intent (curative vs. palliative)
Moderate	Discrepancy across one boundary	*n* = 50 (16.3%)	*n* = 6 (2.0%)	Treatment intensity changes
Low	Match within boundary	*n* = 22 (7.2%)	*n* = 4 (1.3%)	No expected change in treatment plan
None	UICC match	207 (67.6%)		Identical treatment allocation

## Discussion

Despite only moderate overall volumetric overlap the algorithm achieved high lesion-level detection sensitivity across T-, N- and M categories which translated into accurate UICC staging in 67.6% of patients. An additional 8.5% were misclassified within adjacent UICC stages, a discrepancy unlikely to affect management when considered against our predefined clinical decision boundaries. However, clinical relevance of observed errors and particularly false positives in M-staging proved to be highly context-dependent. Overpredictions were predominantly linked to benign or non-oncological pathological findings, which were principal drivers for upstaging. Unlike previous challenge-driven evaluations [5, 7, 19], this study systematically maps segmentation errors to staging outcomes and therapeutic decision boundaries, establishing a blueprint for task-aware benchmarking in real-world oncology workflows. A key contribution of this work is the emphasis on clinical task performance over traditional overlap metrics.

We also observed that linear and complex uptake patterns in pleural and peritoneal regions demonstrated high fidelity which in individual cases outperformed our reference standard highlighting the variability and limitations inherent to expert-defined ground truth [23] and downstream impact on quantification. While predicted T-lesion size fell within the correct T-stage range in more than 95% of patients, size overestimation in the hilar region had a notable impact. Overestimation and undersegmentation in this anatomically complex area led to convergence of central tumours and N1 lymph nodes in 11.1% of cases, highlighting the critical importance of precise delineation in challenging regions.

While overlap-based measures alone may appear insufficient for outcome prediction or treatment planning, lesion-based accuracy metrics and particularly detection sensitivity were encouraging. An exception was M-category precision in the extrathoracic domain, where the broader search space increased the likelihood of false positives. Notably, our error subclassification revealed that the majority of false positives in the M-category were linked to clinically relevant findings rather than physiologic uptake, even if not directly related to tumour burden and TNM staging.

Our study supports the hypothesis that clinical impact of segmentation errors is not uniform across staging tasks and varies in importance depending on the affected TNM component and its contribution to aggregated UICC staging. Some errors are inconsequential for staging purposes, particularly in advanced metastatic disease, while others, especially in early-stage patients, can result in stage migration and potential mismanagement. From a clinical perspective, false positives in M-staging carry the risk of inappropriate treatment escalation. In particular, erroneous classification of extrathoracic lesions as metastases could shift management from curative-intent surgery or radiotherapy towards systemic therapy or palliative concepts. Such overtreatment not only exposes patients to unnecessary toxicity but may also preclude potentially curative interventions. Conversely, our subclassification showed that most false positives were associated with clinically relevant but non-oncological pathologies, which, although driving upstaging, would typically be clarified in multidisciplinary review before treatment decisions are finalised. Although less frequent in our study, the opposite scenario of false negatives may lead to clinically relevant downstaging, for example when a solitary distant metastasis is missed and a patient is incorrectly considered for curative-intent treatment. These observations point to a fundamental trade-off in performance where higher sensitivity may increase the burden of false positives to be resolved in clinical practice, while at the same time reducing the more consequential risk of missing clinically significant disease.

This emphasizes that performance claims must be grounded in clinical tasks and quantified by figures of merit that directly reflect clinical objectives [15, 16, 20, 24, 25]. AI integration spans applications from segmentation to diagnostic support, triage, and ultimately automated TNM staging. As full automation remains several developmental stages away, this study benchmarks current algorithmic performance and explores readiness for clinical integration. Key challenges to clinical implementation include the lack of external validation to address domain shift [26, 27] and the limited integration of human-in-the-loop mechanisms, which present significant opportunities to refine and adapt automated segmentations. To enhance clinical trust and usability, emerging architectures incorporate uncertainty estimation [28, 29], region proposal refinement, and modular pipelines to improve accuracy and interpretability alike. Uncertainty maps for example could help radiologists and nuclear medicine physicians prioritize ambiguous regions, aligning with RELAINCE’s vision of AI as a decision-support tool rather than a black-box replacement.

Several limitations should be considered when interpreting these findings. First, we validated the algorithm based on a task-specific claim in NSCLC patients; broader clinical translation will require prospective, multicentre studies to assess real-world impact of the algorithm on clinical decision-making and patient outcomes. Although our cohort included heterogeneity in patient characteristics and imaging protocols, we did not perform dedicated subgroup analyses due to scope and imbalanced cohort sizes. This remains an area for future investigation. Also, treatment decisions are not based solely on UICC stage but integrate additional factors such as patient performance status, comorbidities, molecular profile, and individual preferences which were not assessed. Second, while the proposed error taxonomy is clinically informed, it remains preliminary and warrants further development, including interobserver calibration and task-specific refinement. Third, our evaluation focused on the highest-performing algorithm from the autoPET III challenge, thereby limiting generalizability across architectures. Consequently, staging relied solely on whole-body [^1^⁸F]FDG PET/CT, without integration of cranial MRI, which contributed to false-negative findings in the detection of brain metastases. Fourth, to mitigate inter-reader variability, ground truth segmentations were established by consensus of two expert readers and multidisciplinary team reference to provide relevant context required for staging which may not be appreciated from imaging alone. In this context, it should be noted that manual lesion delineation was performed without a fixed SUV threshold, which may contribute to variability in total metabolic tumour volume (TMTV) estimation and quantitative assessment. However, as both readers applied consistent anatomical criteria and reached consensus for each case, this effect is unlikely to have influenced lesion detection or staging outcomes. Moreover, no universally accepted standard for manual lesion delineation or SUV-based thresholding in FDG PET/CT exists, which underscores the methodological variability inherent to such quantitative measures. Further potential mitigation strategies could have included additional consensus reads and multiple annotators. Inter-reader variability was not assessed. Also, histopathological confirmation was not available for all equivocal lesions, which may affect the precision of error classification. Finally, oligometastatic disease was not specifically addressed, as it falls outside current TNM and UICC classification frameworks.

## Conclusion

This study demonstrates that despite achieving excellent lesion detection sensitivity (95.8%), the top-performing autoPET III algorithm showed only 67.6% concordance for UICC staging in NSCLC patients, indicating substantial limitations for autonomous deployment. Our task-specific evaluation framework revealed that clinical relevance varied substantially across error types and that upstaging caused by false positive lesions was the primary driver for staging discordance. While systemic overestimation may partly reflect the model’s high sensitivity and its prioritization of not missing clinically relevant lesions, targeted improvements in specificity could significantly enhance its overall clinical utility. Our findings support implementation of diagnostic AI tools as decision-support rather than replacement technology, with mandatory expert oversight for M-stage predictions and cases with multiple lesions. In practice, this could include AI-generated segmentations serving as a first-pass triage to highlight suspicious regions, automated pre-staging reports that are reviewed and corrected by specialists, or uncertainty maps guiding radiologists and nuclear medicine physicians towards ambiguous findings. Future multi-centre studies are warranted to determine how diagnostic AI can be integrated into oncologic hybrid imaging pathways in ways that meaningfully support clinical decision-making.

## Supplementary Information

Below is the link to the electronic supplementary material.

Supplementary file1 (PDF 394 KB)

## Data Availability

The datasets generated during and/or analysed during the current study are available from the corresponding author on reasonable request.
