# Supplementary material for: Artificial intelligence for TNM staging in NSCLC: a critical appraisal of segmentation utility in [1⁸F]FDG PET/CT
Source: Eur J Nucl Med Mol Imaging. 2025 Nov 23;53(5):3117–27. doi: 10.1007/s00259-025-07677-2 (PMC13013355; doi:10.1007/s00259-025-07677-2)
Supplement: Supplementary file 1 — Supplementary file1 (PDF 394 KB) [file 259_2025_7677_MOESM1_ESM.pdf]

	GE Discovery 690	Philips GEMINI TF 16	Philips Vereos PET/CT	SIEMENS Biograph 128 mCT	SIEMENS Biograph 20 mCT	SIEMENS Biograph 40 mCT	SIEMENS Biograph 64 TruePoint
**Standard Activity**	3 MBq /kg bodyweight	3 MBq /kg bodyweight	3 MBq /kg bodyweight	2 MBq /kg bodyweight	3 MBq /kg bodyweight	3 MBq /kg bodyweight	3 MBq /kg bodyweight
**Standard Uptake Time**	60 minutes post injection	60 minutes post injection	60 minutes post injection	60 minutes post injection	60 minutes post injection	60 minutes post injection	60 minutes post injection
**Axial Field of View**	15.7 cm	18.0 cm	25 cm	21.6 cm	16.2 cm	16.2 cm	21.6 cm
**Bed overlap**	23%	50%	50%	23%	23%	23%	23%
**Time per position**	2 min/bed	1.5 min/bed	2 min/bed	2 min/bed	2 min/bed	2 min/bed	2 min/bed
**Matrix**	256 x 256	256 x 256	288 x 288	256 x 256	200 x 200	200 x 200	168 x 168
**Voxel size**	4 x 4 x 3 mm^3	4 x 4 x 4 mm^3	2 x 2 x 2 mm^3	2 x 2 x 2 mm^3	4 x 4 x 2 mm^3	4 x 4 x 2 mm^3	4 x 4 x 3 mm^3
**Reconstruction algorithm**	VPFX with 3 iterations (21 subsets) with TOF	BLOB-OS with 3 iterations (33 subsets) with TOF	BLOB-OS with 3 iterations (20 subsets) with TOF	TrueX with 3 iterations (21 subsets) with TOF	TrueX with 2 iterations (21 subsets) with TOF	TrueX with 3 iterations (24 subsets) with TOF	TrueX with 3 iterations (21 subsets) with TOF
**Applied Corrections**	Attenuation<br>Scatter<br>Randoms<br>Dead-time	Attenuation<br>Scatter<br>Randoms<br>Dead-time	Attenuation<br>Scatter<br>Randoms<br>Dead-time	Attenuation<br>Scatter<br>Randoms<br>Dead-time	Attenuation<br>Scatter<br>Randoms<br>Dead-time	Attenuation<br>Scatter<br>Randoms<br>Dead-time	Attenuation<br>Scatter<br>Randoms<br>Dead-time
**Post Processing Filter**	Gaussian, 6.5 mm FWHM	Gaussian, 6 mm FWHM	Gaussian, 4 mm FWHM	Gaussian, 4 mm FWHM	Gaussian, 3 mm FWHM	Gaussian, 3 mm FWHM	Gaussian, 3 mm FWHM
**CT voltage**	120 kVp	120 kVp	120 kVp	120 kVp	100 kVp	100 kVp	120 kVp
**Tube Current**	auto mA	auto mA	auto mA	auto mA	auto mA	auto mA	auto mA
**Collimation**	64 x 0.625 mm	16 x 1.5 mm	64 x 0.625 mm	64 x 0.6 mm	16 x 1.2 mm	32 x 0.6 mm	64 x 0.6 mm
**Pitch**	1.5	1.0	1.0	1.0	0.8	0.8	0.8

Supplemental Table 1: ***Scanner-specific PET/CT imaging protocols.*** Acquisition parameters for all PET/CT systems used in this study are summarized. Details include scanner model, injected activity, uptake time, axial field of view, bed overlap, acquisition duration, reconstruction algorithm, matrix and voxel size, post-processing filter, and CT acquisition parameters.

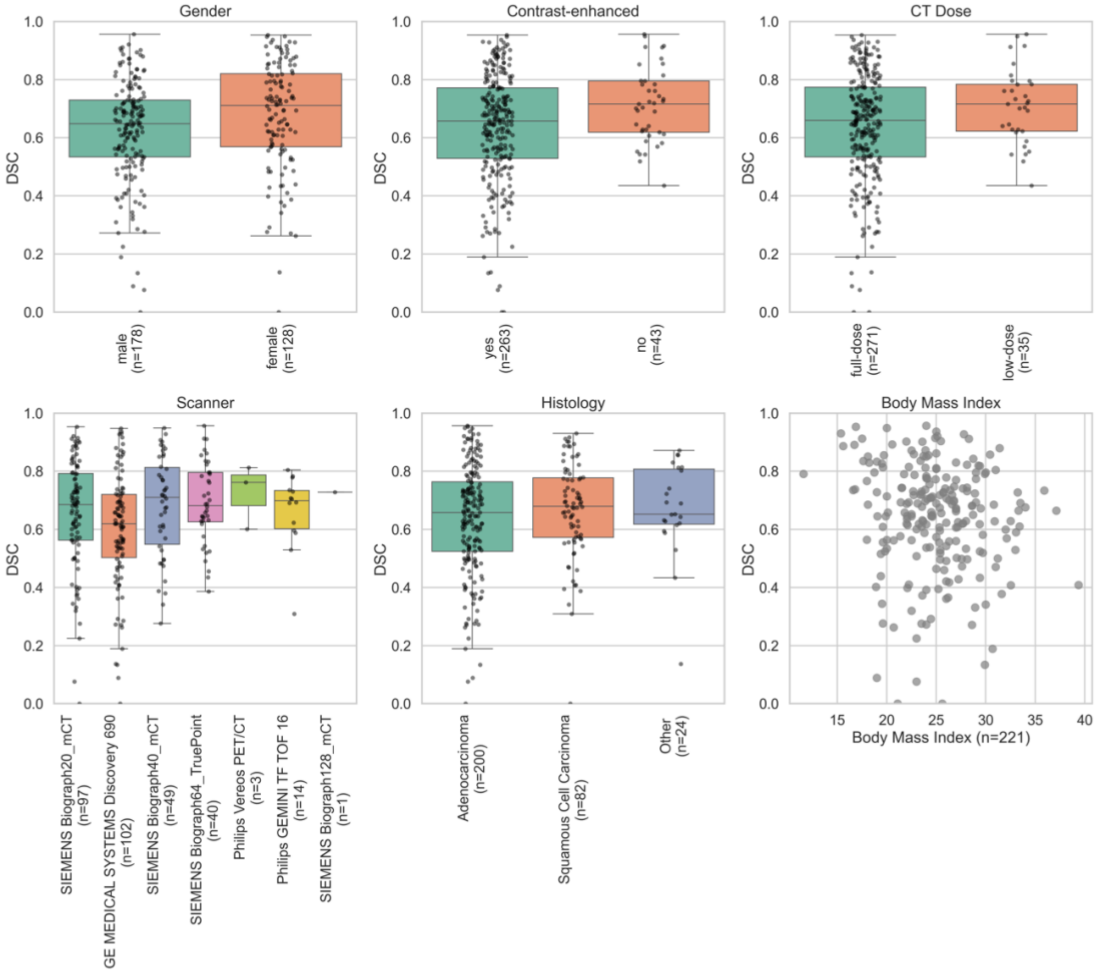

Supplemental Figure 1: Exploratory analysis of model performance (Dice similarity coefficient, DSC) across patient and imaging subgroups. Boxplots show DSC distributions stratified by gender, contrast enhancement, CT dose, scanner type, and histologic subtype; a scatter plot depicts DSC as a function of body mass index (BMI). Performance remained broadly consistent across subgroups, with a mild trend toward higher DSC values in contrast-enhanced and standard-dose CT acquisitions. These results are descriptive and presented as exploratory only, without inferential statistical testing.
